# Reirradiation for Recurrent Nasopharyngeal Carcinomas: Experience From an Academic Tertiary Center in a Low- to Middle-Income Country

**DOI:** 10.1200/JGO.18.00191

**Published:** 2019-02-08

**Authors:** Ryan Anthony F. Agas, Kelvin Ken L. Yu, Paolo G. Sogono, Lester Bryan A. Co, J.C. Kennetth M. Jacinto, Warren R. Bacorro, Michael Benedict A. Mejia

**Affiliations:** ^1^ Benavides Cancer Institute, University of Santo Tomas Hospital, Manila, Philippines

## Abstract

**PURPOSE:**

The objectives of this study were to report the oncologic outcomes and the treatment-related toxicities after reirradiation (re-RT) for recurrent nasopharyngeal carcinoma (rNPC) at our institution and to apply a recently published prognostic model for survival in rNPC in our cohort.

**PATIENTS AND METHODS:**

Thirty-two patients with rNPC treated at the authors' institution with re-RT were retrospectively reviewed. Treatment modalities for re-RT were intensity-modulated radiotherapy (n = 14), three-dimensional conformal radiotherapy (n = 9), single-fraction stereotactic radiosurgery (n = 6), fractionated stereotactic radiotherapy (n = 2), and high dose rate intracavitary brachytherapy (n = 1). Twenty-seven patients received re-RT with curative intent, whereas five patients were treated palliatively.

**RESULTS:**

Median follow-up time was 15.5 months (range, 1 to 123 months) for the entire cohort and 20 months (range, 3 to 123 months) for patients treated with curative intent. For the entire cohort, median locoregional recurrence-free survival (LRRFS) was 14 months, with actuarial 1- and 2-year LRRFS estimates of 67.5% and 44.0%, respectively. Median overall survival (OS) time was 38 months, with actuarial 1- and 2-year estimates of 74.2% and 57.2%, respectively. For patients treated with curative intent, median LRRFS was not reached. Actuarial 1- and 2-year LRRFS estimates were 68.2% and 54.5%, respectively. Median OS time after curative intent re-RT was 42 months, with actuarial 1- and 2-year estimates of 75.4% and 63.8%, respectively. One- and 2-year OS estimates based on risk stratification were 68.6% for high risk compared with 80.8% for low risk and 34.3% for high risk compared with 70.7% for low risk, respectively (*P* = .223). Three patients (9.4%) developed symptomatic temporal lobe necrosis. There was no reported grade 5 treatment-related toxicity.

**CONCLUSION:**

Results of the study suggest that re-RT is an effective and safe salvage treatment strategy for rNPC. Re-RT to a maximum equivalent dose in 2-Gy fractions of 60 Gy may yield good LRRFS and translate to prolonged OS.

## INTRODUCTION

Although contemporary standard management of nasopharyngeal carcinoma (NPC) has improved local control of disease, local failure still remains a concern, especially in advanced T4 disease.^1-3^ Although not proven clinically, three-dimensional conformal radiotherapy (3DCRT) may underdose tumors extending intracranially after field cone-downs to limit toxicity to the optic chiasm or brainstem.^4^ This concern becomes quite relevant in developing countries where conventional radiotherapy (RT) is still being used and where the large majority of patients present with advanced disease.^5^

An accepted approach in the management of recurrent NPC (rNPC) is the delivery of a second round of RT for patients not eligible for nasopharyngectomy.^3,7^ According to a nomogram developed by Riaz et al,^8^ NPC as a primary site fares the best among the other head and neck sites retreated with RT. In patients with good performance status (PS) who receive at least 60 Gy, the 3-year progression-free survival approximates 50%.^9^ A recent publication by Li et al^6^ also reported the development of a prognostic model for survival in patients who undergo salvage reirradiation (re-RT). Utility of this prognostic model may aid in the selection of patients who may benefit from re-RT.^3^

To date, the outcomes of NPC treatment in the Philippines, especially in patients with recurrent disease, have not been reported. This is noteworthy because the Philippines is considered to be one of the countries endemic for NPC.^10,11^ On average, our radiation oncology department treats 30 to 40 new NPCs and three to four rNPCs annually. Given these numbers, our center may be considered a high-volume facility for NPC based on the study by Yoshida et al.^12^ The objectives of this study are (1) to report the oncologic outcomes and treatment-related toxicity after re-RT for rNPC at a tertiary academic center in a low-to middle-income (LMI),NPC-endemic country, and (2) to apply a recently published prognostic model for survival in rNPC in our cohort.^6^

**CONTEXT**

**Key objective:** To our knowledge, this is the first report of reirradiation outcomes for recurrent nasopharyngeal carcinomas from a low- to middle-income, nasopharyngeal carcinoma–endemic country. To our knowledge, it is also the first study to independently apply the prognostic model of Li et al^6^.
**Knowledge generated:** Curative intent reirradiation resulted in good locoregional recurrence-free survival, which translated to prolonged overall survival (OS). The difference in OS between risk groups based on the prognostic model of Li and colleagues failed to reach statistical significance.
**Relevance:** This study shows that even in a low- to middle-income setting, reirradiation for recurrent nasopharyngeal carcinomas, particularly if done in a multidisciplinary fashion, may result in outcomes similar to higher income countries. Application of the Li prognostic model showed similar OS trends between risk subgroups, although this was not statistically significant. Additional investigation with longer follow-up may be needed.

## PATIENTS AND METHODS

This is an institutional review board–approved, retrospective analysis of adult patients with rNPC treated at the Benavides Cancer Institute–University of Santo Tomas Hospital in Manila, Philippines, from 2006 to 2017. All patients were discussed in a multidisciplinary meeting. Patients were determined to have recurrent disease after histologic confirmation (n = 18) or by documented progression by imaging for inaccessible locations (n = 14). Patients deemed to have unresectable disease or who refused surgery were discussed for possible re-RT or chemotherapy depending on the disease extent, age, PS, and symptoms. Patients with nonmetastatic disease and good PS (Eastern Cooperative Oncology Group PS of 0 to 1) were offered salvage re-RT. Given reports of improved outcomes with local RT for select patients with metastatic disease, those with single-organ metastases and good PS were treated to salvage doses up to 60 Gy.^13-17^ Patients with multiple-organ metastases and/or poor PS were treated with palliative treatment (including re-RT). This study reports the outcomes of patients with rNPC treated at our institution with re-RT (salvage or palliative) to the primary and/or regional disease.

Included patients were restaged according to the eighth edition of the American Joint Committee on Cancer classification. Patients with histologies other than undifferentiated or squamous cell carcinomas and who received nonstandard primary treatment were excluded. Re-RT techniques used included 3DCRT, intensity-modulated RT (IMRT), single-fraction stereotactic radiosurgery (SRS), fractionated stereotactic RT (FSRT), or high dose rate (HDR) intracavitary brachytherapy (ICBT).

Computed tomography scan–based 3DCRT was delivered via lateral and anteroposterior fields or wedged-pair techniques. Inverse-planned IMRT was given using the step-and-shoot delivery mode. Both external-beam RT techniques used the Philips Pinnacle Treatment Planning system (TPS) version 7.6c (Philips, Andover, MA). Dose was prescribed to the high-risk planning target volume (PTV), which was defined as the recurrent gross tumor volume (rGTV) plus a 0.5- to 1-cm clinical target volume margin, plus an additional 0.5-cm PTV margin. Almost all patients treated with curative intent were prescribed a total dose of 60 Gy in 2-Gy daily fractions. One patient received hyperfractionated RT to a total dose of 64.96 Gy in 1.12-Gy twice-daily fractions (60.2 Gy equivalent dose in 2-Gy fractions [EQD2]) because of the proximity of the rGTV to neural structures and the relatively short interval (6 months) between primary treatment and re-RT. Ideal prescription plans were to have at least 95% of the high-risk PTV within 95% of the prescription dose. Although data on primates suggest substantial recovery of neural structures after 2 years from RT, the authors assumed a more conservative value of, at most, half dose tolerance recovery (Table 1).^18^ Patients treated with external-beam RT for palliation received either 20 Gy in five fractions or 30 Gy in 10 fractions.

**TABLE 1:**
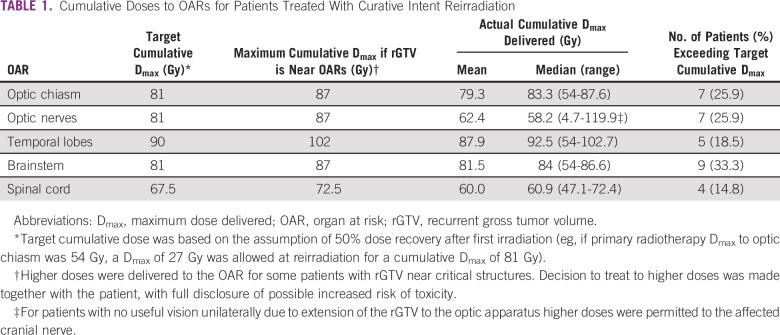
Cumulative Doses to OARs for Patients Treated With Curative Intent Reirradiation

Patients with early (rT1-2) and/or nonbulky (≤ 4 cm maximum diameter) recurrent tumors were considered for SRS, FSRT, or HDR ICBT. SRS and FSRT were delivered via frame-based radiosurgery techniques using the Radionics XKnife RT version 4.0.1 TPS (Integra LifeSciences, Plainsboro, NJ). SRS (n = 6) was delivered to a median dose of 16.5 Gy (range, 12 to 18 Gy), whereas the FSRT (n = 2) total dose was 24.4 Gy in five fractions. Both SRS and FSRT were prescribed to the 80% isodose line. The decision to treat with either SRS or FSRT depended on proximity to neural structures (FSRT preferred if rGTV was within 3 mm) and the tumor size (FSRT preferred for tumors > 3 cm up to 4 cm in largest diameter). HDR ICBT was delivered using an iridium-192–based, microSelectron Nucletron (Elekta, Stockholm, Sweden) stepping source. Balloon applicators with two nasopharyngeal catheters were inserted at each nostril. On the basis of the diagnostic magnetic resonance imaging (MRI), the target volume was determined on orthogonal films. Using the Nucletron Genie TPS version 1.0.3 (Nucletron, Columbia, MD), a prescription dose of 21 Gy in three fractions was prescribed to 1 cm from the axis of the applicators. We used the points for monitoring organs at risk as defined by Levendag et al.^19^

On the discretion of the attending physicians, systemic therapy was given concurrently and/or as induction to re-RT. Cisplatin-based chemotherapy regimens were preferred for eligible patients (acceptable renal function, < 600 mg/m^2^ prior cumulative cisplatin dose, and without clinically significant hearing loss). Otherwise, patients received either concurrent carboplatin with fluorouracil or concurrent cetuximab or were treated with re-RT alone. Patients with advanced recurrent nodal disease (rN3) or advanced recurrent primary tumor (rT4) were evaluated for possible induction chemotherapy.

Clinic follow-up with nasopharyngeal endoscopy was conducted at least every 3 months for the first 2 years, every 6 months until 5 years, and then annually thereafter. The preferred imaging modality for surveillance was gadolinium-enhanced MRI with diffusion-weighted imaging. Ideally, surveillance MRI was obtained 3 to 4 months after re-RT, every 6 to 8 months for the first 2 years, and then annually thereafter. All patients were observed at our institution.

The primary outcomes were locoregional recurrence-free survival (LRRFS) and overall survival (OS). LRRFS was defined as the proportion of patients alive without local and/or regional recurrence at a specified period from the date of initiation of re-RT. OS was defined as the proportion of patients alive after a specified period from the date of initiation of re-RT. Secondary outcome measures were acute and late treatment-related toxicities, which were scored according to the Radiation Therapy Oncology Group radiation morbidity grading and the Common Terminology Criteria for Adverse Events version 5.0, respectively.^20,21^

Univariate analysis of prognostic factors was conducted for patients treated with curative intent. We used cutoffs for age and recurrent tumor volume used by prior studies, which reported these as significant prognosticators.^22-25^ To determine patterns of failure after primary RT, we fused the primary and re-RT plans. This was done via rigid registration based on bony landmarks (top of the dens, C1 to C2 vertebrae, pterygoid plates, hard palate, and the clinoid processes). The doses received during primary RT by the rGTV were determined using dose-volume histograms. Because this required having both the primary and re-RT plans in our TPS, this analysis was only conducted for patients who had primary RT at the home institution. We used the definition for patterns of failure as published in the literature (in field: at least 95% of rGTV within 95% of prescription dose; marginal: < 95% but not < 20% of rGTV within 95% of prescription dose; out of field: < 20% of rGTV within 95% of prescription dose).^26-29^ For eligible patients (nonmetastatic, curative intent treatment), we applied the prognostic model for OS by Li et al.^6^ This model uses several covariates (ie, age, rGTV, prior grade 3 toxicities, rT stage, and re-RT EQD2) to classify patients as high or low risk (Table 2).

**TABLE 2:**
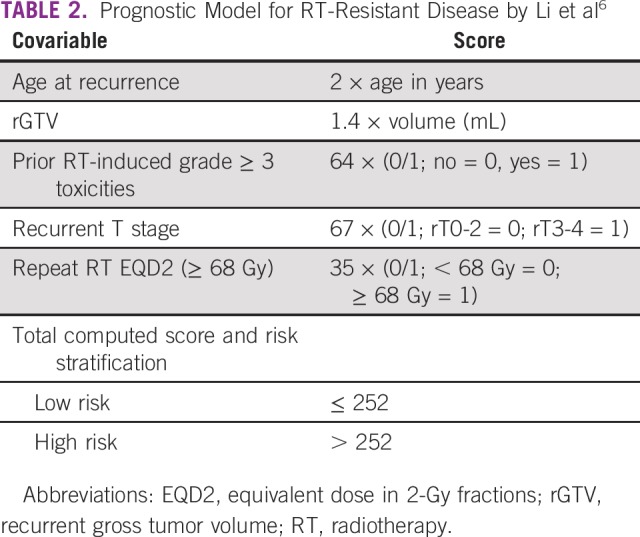
Prognostic Model for RT-Resistant Disease by Li et al^6^

Data analysis was done using SPSS Statistics version 24 (SPSS, Chicago, IL). Categorical variables were tested using the χ^2^ test. Missing data were handled via listwise deletion. Actuarial OS and LRRFS were calculated using the Kaplan-Meier method. Univariate analysis of prognostic factors was performed using log-rank tests. We considered *P* < .05 as statistically significant.

## RESULTS

### Patient and Treatment Characteristics

A total of 32 patients were included in our cohort. Patient and treatment characteristics are listed in Table 3. Median time to first recurrence was 22 months (range, 6 to 120 months) from completion of primary RT, with 80% of recurrences occurring within 43 months.

**TABLE 3:**
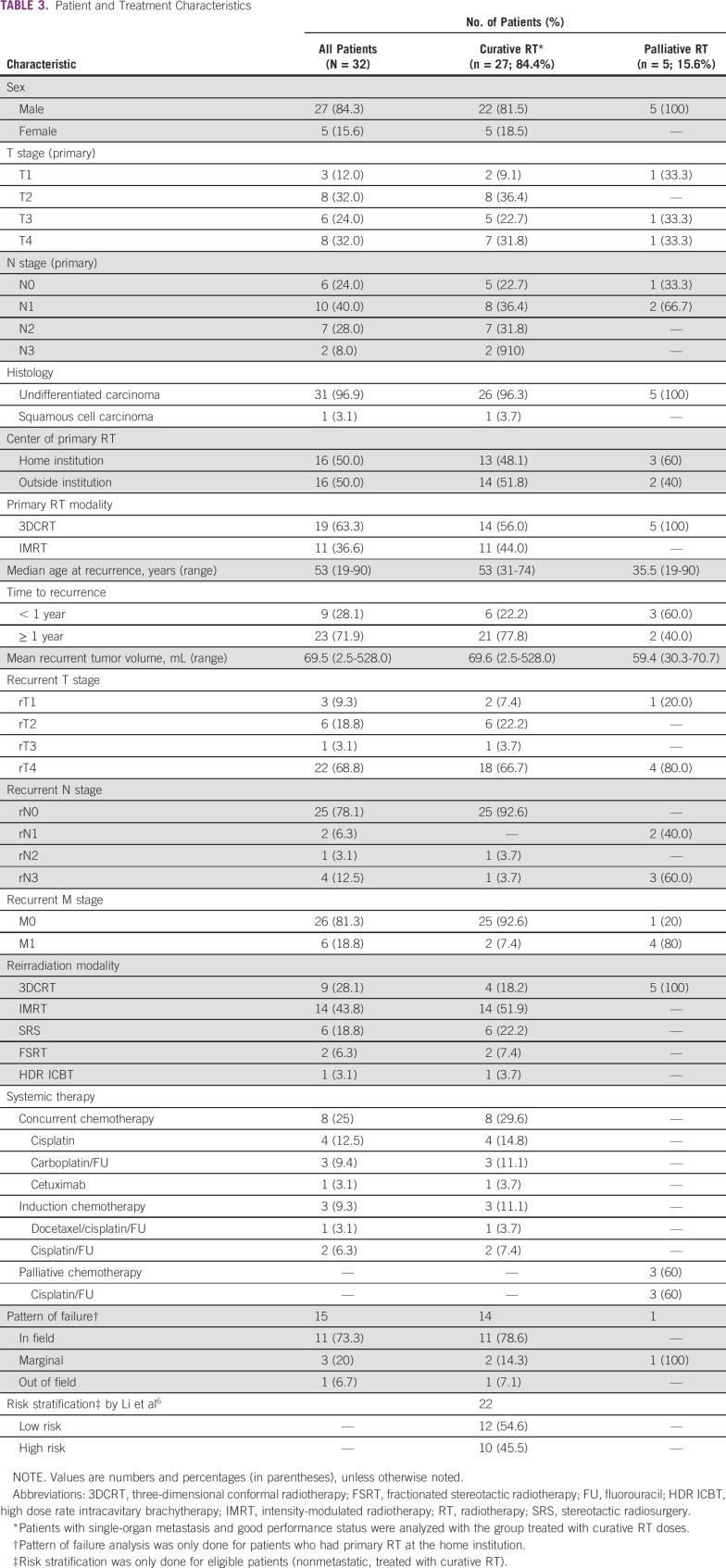
Patient and Treatment Characteristics

### Oncologic Outcomes

**FIG 1:**
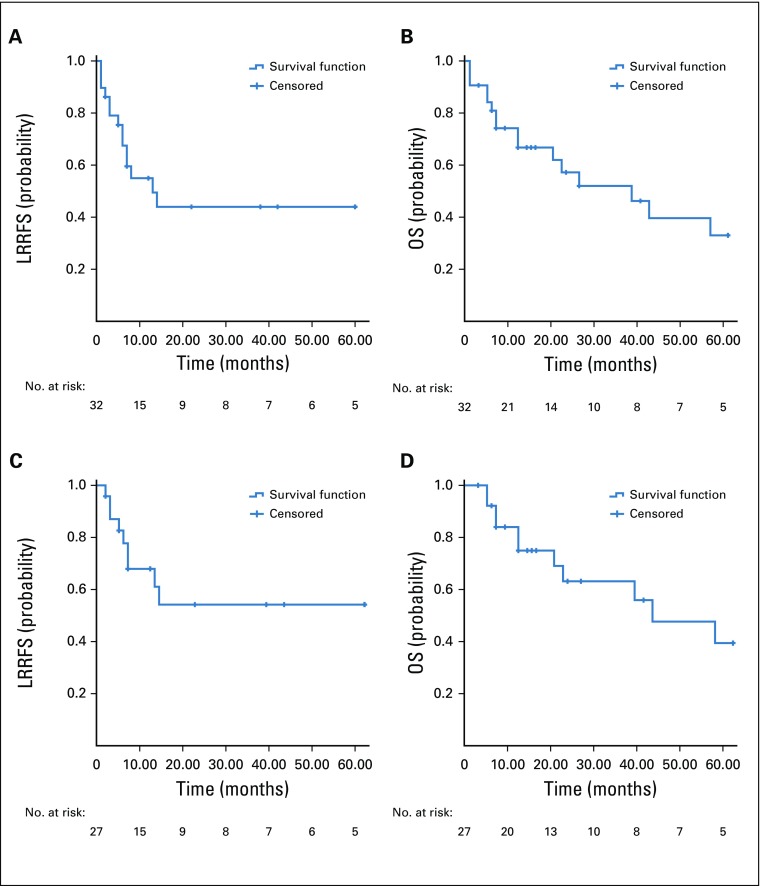
Oncologic outcomes. (A) Actuarial locoregional recurrence-free survival (LRRFS) of the entire cohort. (B) Actuarial overall survival (OS) of the entire cohort. (C) Actuarial LRRFS of patients treated with curative intent. (D) Actuarial OS of patients treated with curative intent.

Median follow-up time for the entire cohort was 15.5 months (range, 1 to 123 months). Median LRRFS was 14 months (range, 3.9 to 22.0 months), with actuarial 1-, 2-, and 3-year LRRFS estimates of 67.5%, 44.0%, and 44.0%, respectively (Fig 1A). Median OS for the entire cohort was 38 months (range, 14.6 to 61.4 months), with actuarial 1-, 2-, and 3-year OS estimates of 74.2%, 57.2%, and 52.1.%, respectively (Fig 1B).

For patients treated with curative intent (median follow-up, 20 months; range, 3 to 123 months), the median LRRFS was not reached. Actuarial 1-, 2-, and 3-year LRRFS estimates for these patients were 68.2%, 54.5%, and 54.5%, respectively (Fig 1C). Median OS for this group was 42 months, with actuarial 1-, 2-, and 3-year OS estimates of 75.4%, 63.8%, and 63.8%, respectively (Fig 1D).

Palliatively treated patients had a median OS of 6 months (range, 1 to 24 months), with a 1-year actuarial OS estimate of 30%. All five patients had pain as the primary complaint, with 80% of patients (four of five patients) reporting decreased pain score on post-treatment follow-up.

**FIG 2:**
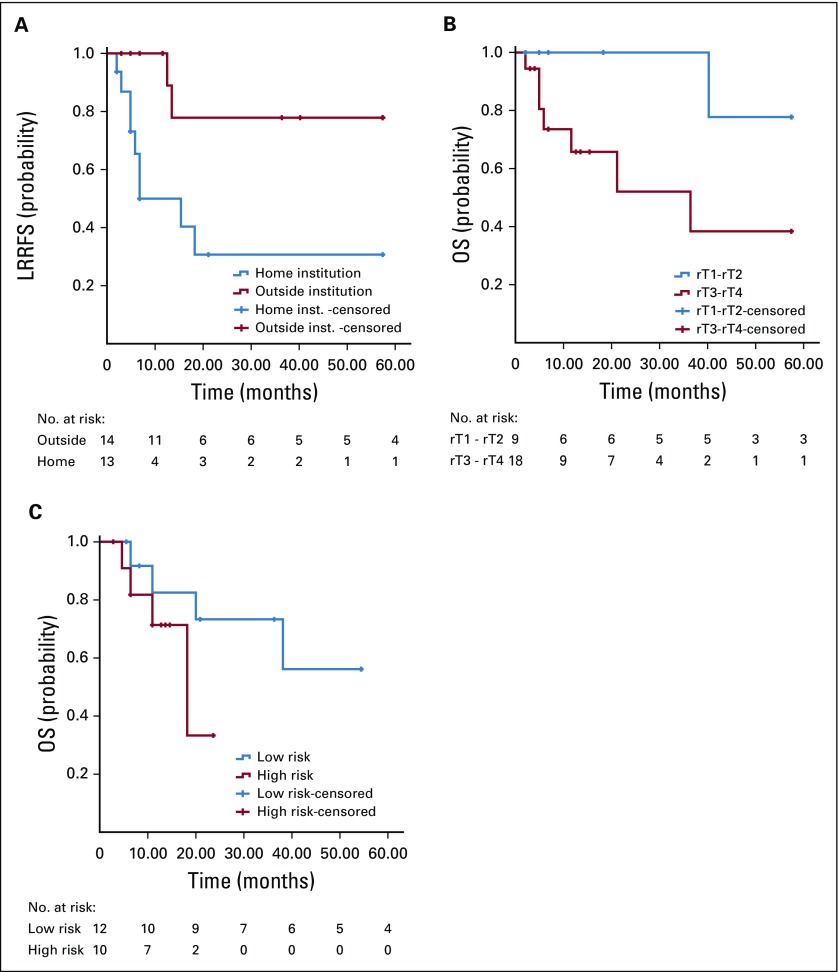
Univariable analysis. (A) Locoregional recurrence-free survival (LRRFS) of patients treated with curative intent according to center of primary radiotherapy (home institution (inst.) *v* outside institution; *P* = .012). (B) Overall survival (OS) of patients treated with curative intent according to recurrent T stage (rT1-2 *v* rT3-4; *P* = .01). (C) OS of patients treated with curative intent according to risk classification (low *v* high risk; *P* = .22).

On univariable analysis (Table 4), primary RT at the home institution (*v* an outside institution) was associated with poorer LRRFS (*P* = .012; Fig 2A). Advanced rT stage (rT3-4 *v* rT1-2 disease) was associated with poorer OS (*P* = .017; Fig 2B). Analysis based on the prognostic model by Li et al^6^ showed that the OS difference between high-risk and low-risk patients (1-year OS, 68.6% *v* 80.8%, respectively; 2-year OS, 34.3% *v* 70.7%, respectively) failed to reach statistical significance (*P* = .223; Fig 2C).

**TABLE 4:**
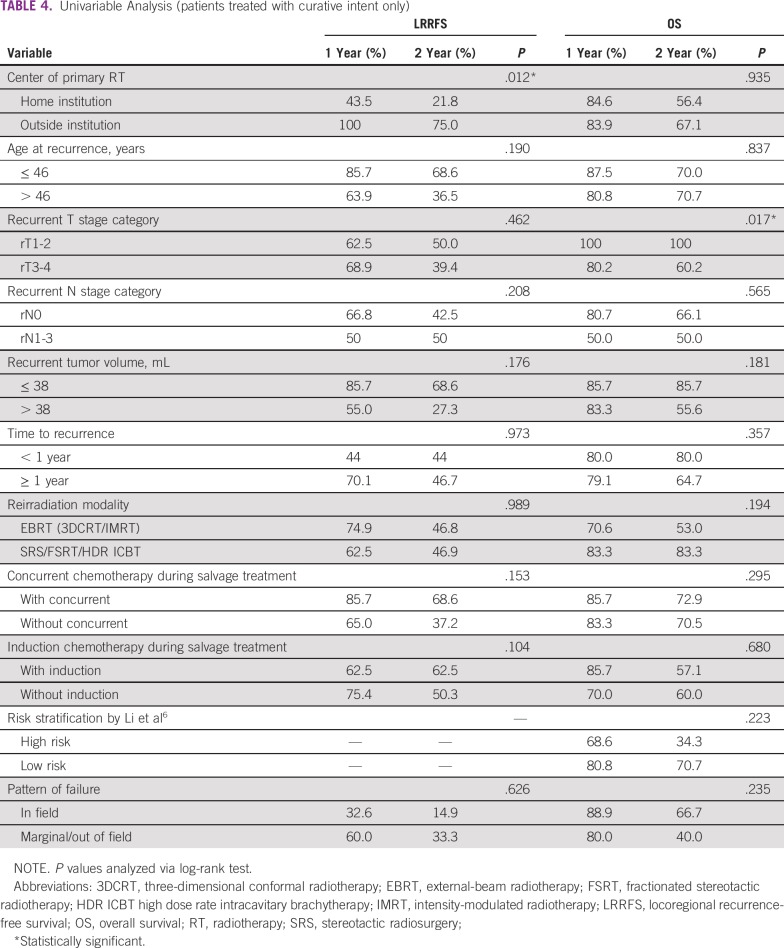
Univariable Analysis (patients treated with curative intent only)

### Treatment-Related Toxicity

Cumulative incidences of acute grade 1 to 2 and grade 3 to 4 toxicity were 25.0% and 6.5%, respectively. Grade 1 or 2 acute toxicities included mucositis (n = 5, 15.6%) and esophagitis (n = 3, 9.3%). Severe (grade 3) acute mucositis occurred in one patient (3.1%), whereas another patient (3.1%) developed grade 3 dermatitis. One patient (3.1%) developed grade 1 Lhermitte syndrome 4 months after completion of re-RT. Twenty-three percent of patients developed severe late toxicities, but there was no documented treatment-related mortality. Three patients (9.4%) developed late symptomatic temporal lobe necrosis. Five patients (15.6%) had new-onset cranial neuropathy, which was managed conservatively. These neuropathies occurred at a median of 9 months (range, 6 to 24 months) after completion of re-RT. Cranial neuropathies presented as ipsilateral facial numbness (n = 2), worsening of visual acuity (n = 2), and diplopia (n = 1). Average maximum cumulative dose to the optic apparatus for patients with presumed optic neuropathy was 85.7 Gy (median, 85.6 Gy; range, 84.7 to 86.8 Gy), which was higher than the target cumulative dose (Table 1).

## DISCUSSION

To our knowledge, this is the first report of outcomes after re-RT for rNPC in the Philippines, which is an LMI, NPC-endemic country. The majority of the reported studies come from countries belonging to higher income groups and/or from nonendemic countries.^24,29-36^ Results of our study seem to concur with evidence that indicate that re-RT should be considered a valuable salvage option for patients with rNPC (Table 5).^7,37,38^ Although our re-RT doses were more conservative compared with some of the published studies, our outcomes suggest that re-RT to a maximum EQD2 of 60 Gy may result in good locoregional control of disease and translate to prolonged survival. These findings may also imply that even in an LMI setting, retreatment done in a multidisciplinary fashion may result in outcomes similar to those seen in higher income countries.

**TABLE 5:**
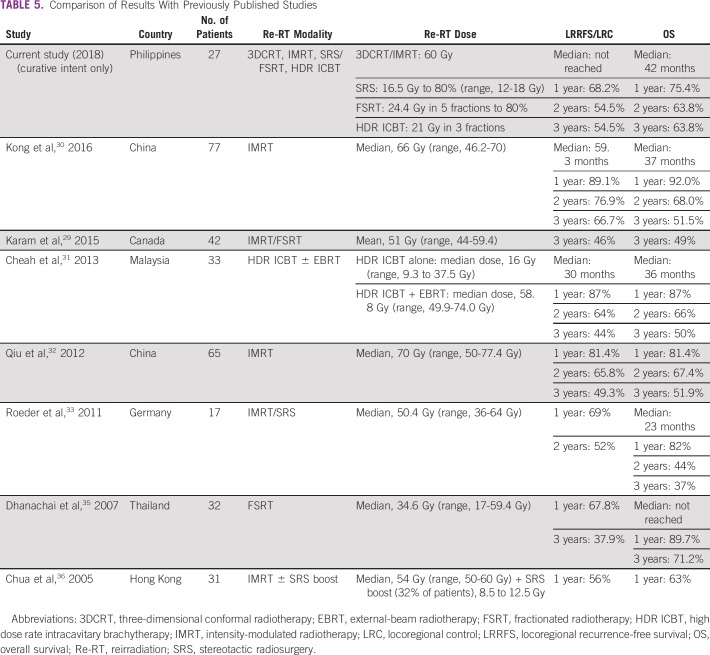
Comparison of Results With Previously Published Studies

Compared with our cohort, some contemporary studies have reported relatively higher incidences of severe late toxicity after re-RT, including treatment-related mortality.^6,23,30^ A common feature of these studies is the delivery of relatively high re-RT doses (66 to 70 Gy). However, published studies that prescribed re-RT to lower doses did not report any grade 5 toxicity.^29,33^ Evidently, there seems to be a cumulative dose-response relationship with severe late toxicity. This needs to be balanced with the possible need for higher re-RT doses for more advanced, RT-resistant disease. Currently, no standard maximum threshold dose for re-RT is established, but the prognostic model by Li et al^6^ suggests that re-RT doses of 68 Gy or greater may increase the risk for grade 5 toxicity. Yu et al^39^ also suggested that a cumulative gross tumor volume dose of 141.5 Gy or greater may increase the risk of lethal nasopharyngeal necrosis.

The publication by Li et al^6^ reported a prognostic model for rNPC based on cohorts from China and Singapore. To our knowledge, this is the first study (particularly from an LMI country) to independently use that prognostic model and report outcomes. Applying their prognostic tool, the difference in OS between our risk subgroups failed to reach statistical significance (*P* = .2235). However, similar to the results in their study, our survival curves started to separate after 1 to 2 years (Fig 2C). The lack of a statistically significant difference could have been the result of the relatively small number of patients (n = 22) in this analysis. Longer follow-up may also be needed to adequately assess the applicability of the prognostic model in our cohort.

**FIG 3:**
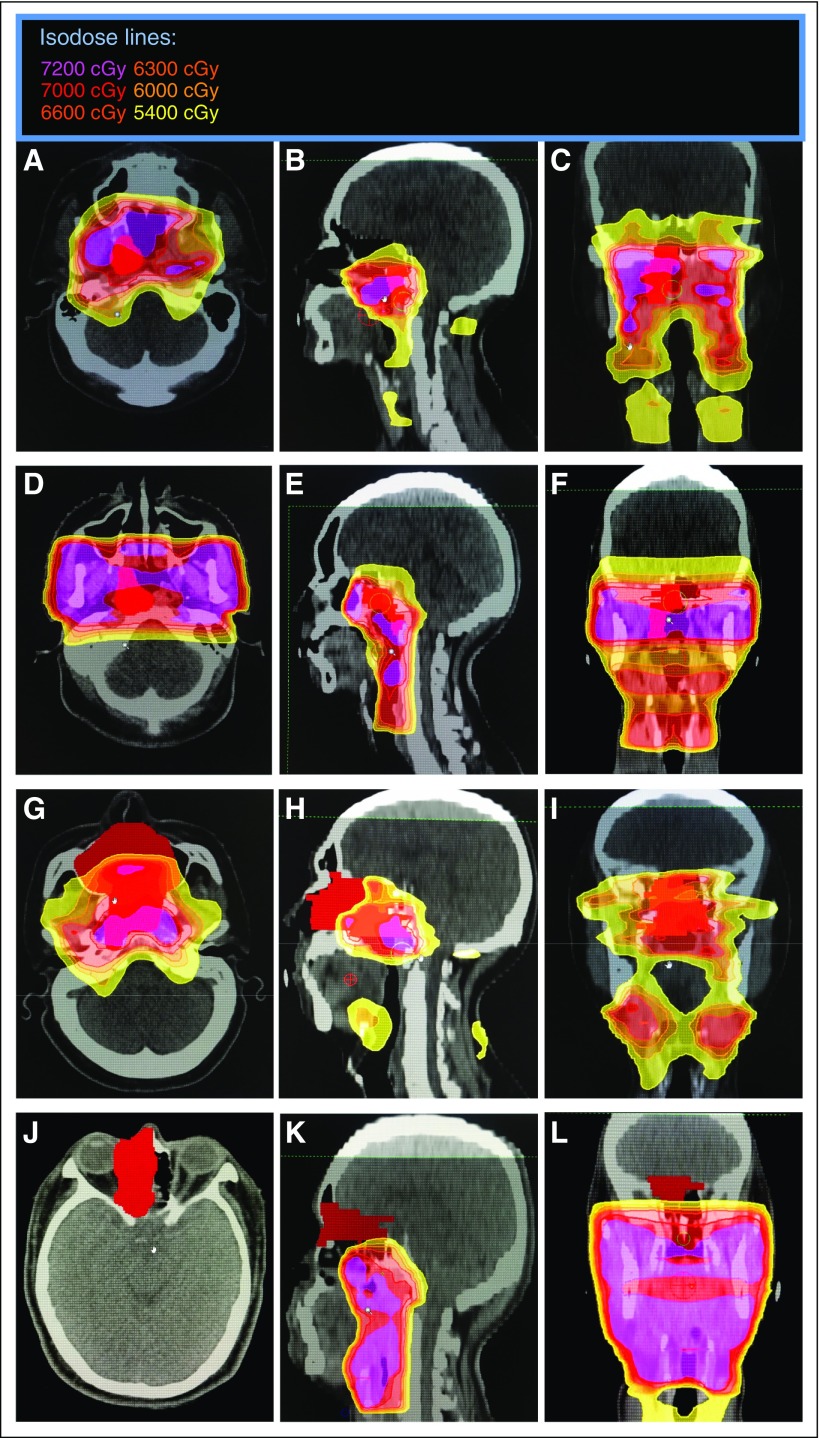
Primary radiotherapy (RT) at the home institution. (A to C) Representative cuts of the primary RT computed tomography (CT) simulation plan of a patient who had primary RT (intensity-modulated RT [IMRT]) at the home institution with an in-field failure. (D to F) Representative cuts of the primary RT CT simulation plan of another patient who had primary RT (three-dimensional conformal RT [3DCRT]) at the home institution with an in-field failure. (G to I) Representative cuts of the primary RT CT simulation plan of the patient who had primary RT (IMRT) at the home institution and had a marginal failure. (J to L) Representative cuts of the primary RT CT simulation plan of the patient who had primary RT (3DCRT) at the home institution and had an out-of-field failure. Tumor volume in solid red indicates recurrent gross tumor volume contoured on the primary RT CT simulation plans.

Our univariable analysis showed that rT stage was a significant predictor for OS. This is in line with other studies reporting poorer survival in patients with advanced rT stage.^23-25,29,30,32,33^ The analysis also showed that patients who had primary treatment at the home institution had significantly worse LRRFS compared with patients initially treated at outside institutions. Dose-volume histogram analysis of re-RT plans showed no significant difference in dosimetric parameters between the two groups (Table 6). Interestingly, analysis of patterns of failure after primary RT showed that 84.6% of the patients (11 of 13 patients) treated with curative intent re-RT at the home institution had in-field recurrences. Examples of these in-field recurrences can be seen in Figures 3A-3F. The other two failures were a marginal recurrence (Figs 3G-3I) and an out-of-field recurrence (Figs 3J-3L).

**TABLE 6:**
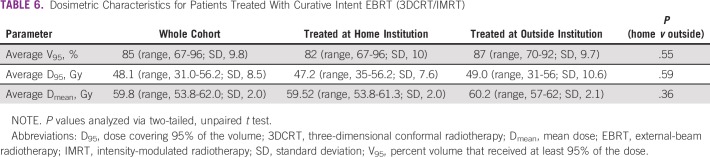
Dosimetric Characteristics for Patients Treated With Curative Intent EBRT (3DCRT/IMRT)

**FIG 4:**
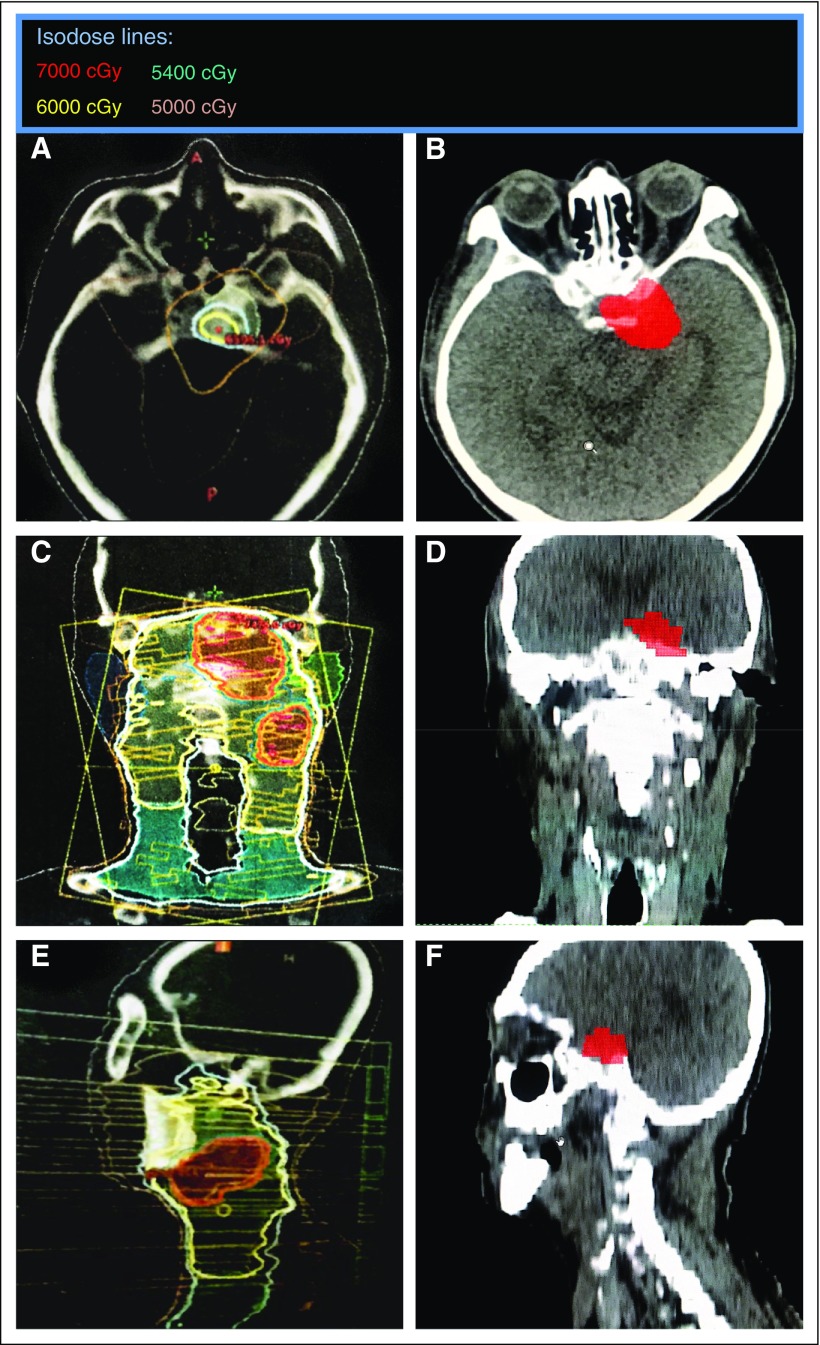
Primary radiotherapy (RT) at an outside institution. (A to C) Representative cuts of the primary RT computed tomography (CT) simulation plan of a patient who had primary RT (RapidArc intensity-modulated RT; Varian Medical Systems, Palo Alto, CA) at an outside institution. (D to F) Comparative cuts of the reirradiation (re-RT) CT simulation images of the same patient who had primary RT at an outside institution. Tumor volume in solid red indicates recurrent gross tumor volume contoured on the re-RT CT simulation plan.

On the basis of our observations, there could have been more patients who had primary treatment at outside institutions with marginal or out-of-field recurrences after primary RT. Figures 4A-4F show an example of one such patient who had primary RT from an outside institution. Comparison of the rGTV with the primary RT isodose curves available suggests dosimetric inadequacy within the region of recurrence. In addition, six other patients who had primary RT at outside institutions were also presumed to have marginal or out-of-field recurrences. We would like to emphasize that this observation regarding patients who had primary treatment at outside institutions was strictly based on conjecture, because their primary RT plans were not available in our TPS. Nevertheless, if the observations were accurate, there would seem to be a trend (*P* = .056) toward more marginal and out-of-field treatment failures in patients who had primary treatment at outside institutions (seven [50%] of 14 patients) compared with patients who had primary treatment at the home institution (two [15.3%] of 13 patients). This occurred despite having six of the seven marginal or out-of-field failures from outside centers treated with primary IMRT. A possible reason for this may be the differences in facility volume between the home and outside institutions. In fact, two of the patients with marginal or out-of-field treatment failures had primary IMRT done in nonendemic countries.

Combining our dosimetric analysis and observations regarding patterns of failure after primary RT could partly explain the difference in LRRFS between the two primary RT center subgroups. Patients with in-field treatment failures after primary RT may be assumed to have true RT-resistant disease.^40^ Although not proven clinically, these patients may be expected to respond worse to re-RT compared with patients who had either marginal or out-of-field recurrences. Should patients with in-field, RT-resistant disease be considered for intensification of salvage treatment, or should they be considered for other non-RT modalities? The answer to this question is beyond the scope of this article but may be worth investigating in future studies.

Although there have been several reports of palliative RT for incurable head and neck cancers, data are limited regarding palliative re-RT for rNPCs. Albeit a small sample only, our survival outcomes for patients treated with palliative re-RT are comparable to those in studies using RT for palliation in other head and neck primary tumors. Similar to our cohort, the studies by Porceddu et al,^41^ Das et al,^42^ and Corry et al^43^ reported median survival times of 6 months, 7 months, and 5.7 months, respectively. Improvement in quality-of-life scores has also been previously reported in 44% to 100% of patients who had RT with palliative intent.^41,43,44^ Although we were not able to report quality-of-life measures for our cohort, pain score was decreased in 80% of our patients.

This study is limited by its retrospective nature. Some patients included in this study had missing data, which could have affected the analysis. Longer patient follow-up may be needed to more adequately evaluate the late toxicities. The study also had a relatively small sample size, which may limit the interpretation of prognostic factors. Dosimetric analysis of patterns of failure and cumulative organ at risk dose may have been limited by the use of rigid registration techniques, which has been reported to be inferior to deformable methods.^45-47^

In conclusion, results of the study suggest that re-RT is an effective and safe salvage treatment strategy for rNPC. Re-RT to a maximum EQD2 of 60 Gy may result in long-term LRRFS and OS. The difference in OS between risk groups based on the prognostic model of Li et al^6^ failed to reach statistical significance.
